# Retrospective analysis of central venous catheters in elective intracranial surgery - Is there any benefit?

**DOI:** 10.1371/journal.pone.0226641

**Published:** 2019-12-19

**Authors:** Benjamin Löser, Olga Recio Ariza, Alexander März, Anastassia Löser, Jörn Grensemann, Martin Petzoldt, Daniel A. Reuter, Frank Weber, Änne Glass, Sebastian A. Haas

**Affiliations:** 1 Department of Anaesthesiology, Center of Anaesthesiology and Intensive Care Medicine, University Medicine Rostock, Rostock, Germany; 2 Department of Anaesthesiology, Center of Anaesthesiology and Intensive Care Medicine, University Medical Center Hamburg-Eppendorf, Hamburg, Germany; 3 Department of Radiotherapy and Radiation Oncology, University Medical Center Hamburg-Eppendorf, Hamburg, Germany; 4 Department of Intensive Care Medicine, Center of Anaesthesiology and Intensive Care Medicine, University Medical Center Hamburg-Eppendorf, Hamburg, Germany; 5 Institute for Biostatistics and Informatics in Medicine and Ageing Research, University Medicine Rostock, Rostock, Germany; Cleveland Clinic, UNITED STATES

## Abstract

**Background:**

It remains unclear whether the use of central venous catheters (CVC) improves a patient's clinical outcome after elective intracranial supratentorial procedures.

**Methods:**

This two-armed, single-center retrospective study sought to compare patients undergoing elective intracranial surgery with and without CVCs. Standard anaesthesia procedures were modified during the study period resulting in the termination of obligatory CVC instrumentation for supratentorial procedures. Peri-operative adverse events (AEs) were evaluated as primary endpoint.

**Results:**

The data of 621 patients in total was analysed in this study (301 with and 320 without CVC). Patient characteristics and surgical procedures were comparable between both study groups. A total of 132 peri-operative AEs (81 in the group with CVC vs. 51 in the group without CVC) regarding neurological, neurosurgical, cardiovascular events and death were observed. CVC patients suffer from AEs almost twice as often as non CVC patients (OR_adjusted_ = 1.98; 95%CI[1.28–3.06]; p = 0.002). Complications related to catheter placement (pneumothorax and arterial malpuncture) were observed in 1.0% of the cases. The ICU treatment period in patients with CVC was 22 (19;24) vs. 21 (19;24) hours (p = 0.413). The duration of hospital stay was also similar between groups (9 (7;13) vs. 8 (7;11) days, p = 0.210). The total time of ventilation (350 (300;440) vs. 335 (281;405) min, p = 0.003) and induction time (40 (35;50) vs. 30 (25;35) min, p<0.001) was found to be prolonged significantly in the group with CVCs. There were no differences found in post-operative inflammatory markers as well as antibiotic treatment.

**Conclusion:**

The data of our retrospective study suggests that patients undergoing elective neurosurgical procedures with CVCs do not demonstrate any additional benefits in comparison to patients without a CVC.

## Introduction

More than five million central venous catheters (CVC) are placed in the United States per year.[1] CVCs facilitate many medical procedures such as catecholamine infusion, administration of intravenous medication, parenteral nutrition, acquisition of blood samples and monitoring of central venous pressure. Furthermore, CVCs are also used for transpulmonary thermodilution measurements.[2] Each and any CVC insertion requires precise medical indications in order to prevent unnecessary risks and complications. Nevertheless, ultrasound-guided CVC insertion has reduced complications and qualitatively increased the safety of CVC insertions.[3] CVCs are associated with numerous risks and side effects.[4–9] Vascular complications including venous injury, arterial puncture / cannulation as well as hemorrhage, haematoma and haemathorax may occur. Furthermore, pneumothoraces, pneumomediastinum, tracheal injury, nerve lesions (e.g. phrenic nerve) and chylothorax are examples of other possible adverse events (AEs). Cardiac events such as arrhythmia, cardiac arrest and tamponade due to ventricular perforation, thrombosis and thrombophlebitis must be taken into account. Catheter related blood stream infections are also a feared complication and frequently lead to sepsis, septic shock and even death. It is due to these potential complications that measures have been taken in order to develop less invasive alternatives, in order to facilitate blood sampling, drug administration and hemodynamic monitoring without the need of a CVC.[10] Some authors believe that CVCs will be deemed obsolete in the future.[10]

The anaesthesia management of patients undergoing elective intracranial supratentorial surgery varies between clinical institutions as no standardized recommendations or guidelines have been defined. Most institutions coincide in the implementation of general anaesthesia and continuous blood pressure monitoring by means of an arterial line during intracranial procedures. However, the use of central venous lines is deemed controversial [11–14] as their clinical benefit remains unclear for patients undergoing intracranial surgery.

The primary endpoint of this study was peri-operative and post-operative AEs which were previously defined and compared between groups. The secondary endpoints included clinical outcome parameters such as procedural AEs and hospital stay. Inflammatory parameters (CRP and leukocytes) during the ICU stay were also analysed. Furthermore, ICU treatment duration and further peri-operative times such as anaesthesia induction time and total time of ventilation were determined.

## Material and methods

The Ethics Committee of the Medical Board of Hamburg, Germany approved this single-center retrospective study (WF 067/17) on the 4^th^ of December 2017. This study was carried out at the University Medical Center Hamburg-Eppendorf, a hospital with 1400 beds providing maximum care. Patient data was collected from June 2016 to January 2018 and retrospectively analysed. The data acquisition was finalised after the patient was transferred from the ICU to a peripheral ward. Anaesthesia records were screened for patient characteristics such as gender, age, ASA (American Society of Anesthesiologists) classification, body mass index and relevant co-morbidities. Information regarding ICU treatments and other relevant data were obtained from anaesthesia records as well as from the following in-house data management programs: Integrated Care Manager (ICM) (Release 9.1, Dräger, Lübeck, Germany) and Soarian™ Clinicals (Release 4.01, SP08, Siemens Healthcare, Erlangen, Germany). This data was then plotted into an excel sheet (Microsoft Excel, Release 2010) for further statistical analysis.

### Inclusion criteria

The inclusion criteria were defined to be: ≥ 18 years, ASA I-IV, elective intracranial supratentorial surgery and a scheduled post-operative ICU admission.

### Peri-operative and procedural AEs

Peri-operative complications were pre-defined prior to the initiation of this study. In order to assess peri-operative AEs, anaesthesia records as well as daily examination protocols and any other ICU documentation in electronic form were screened for any abnormalities. Furthermore, patient data was screened for neurological, neurosurgical and any cardiovascular AEs. Procedural AEs were determined to be pre-operative CVC placement complications such as pneumothorax, arterial malpuncture or unsuccessful / multiple venous punctures.

### Anaesthesia management and intensive care unit

All patients undergoing elective supratentorial surgery were prepared according to institutional standards. In certain cases (e.g. high-grade glioma) patients did however, receive a high dose of steroids pre-operatively.[15] The analysed patients neither suffered from a severe (untreated) coagulopathy nor necessitated a peri-operative therapeutic anticoagulation treatment during this study. All patients undergoing elective supratentorial surgery were prepared according to institutional standards. The anaesthesia induction was carried out using Remifentanil (0.2–0.5 μg/kg/min) or Sufentanil (0.2–0.5 μg/kg) and Propofol (1.5–2.5 mg/kg). Rocuronium (0.6–1 mg/kg) was used as the standard muscle relaxant in order to facilitate endotracheal intubation. Anaesthesia was maintained using the following intravenous medication: Propofol (6–8 mg/kg/h) and Remifentanil (0.2–0.5 μg/kg/min). A Primus® (Dräger) ventilation device was used after endotracheal intubation. Standard monitoring consisted of a three-lead electrocardiogram, oscillometric noninvasive blood pressure monitoring, side-stream capnography, pulse oximetry as well as continuous invasive blood pressure monitoring.

The method of CVC placement during the study period (landmark vs. ultrasound guided) was left to the discretion of the anaesthesiologist. The site was cleaned prior to the puncture of the corresponding vein. A hollow needle was advanced through the skin until blood was able to be aspirated. A guide wire was introduced via the hollow needle after verifying the position of the needle (color and flow of the blood and / or via ultrasound). Afterwards, the needle was removed and a dilating device was introduced before the triluminal, non-heparin covered central venous line was passed over the guidewire before removing the wire. An aspiration test was performed through all lumens. Furthermore, in order to ensure the placement of the catheter in the vein, venous pressure was measured prior to the administration of drugs. The catheter was then flushed with a 0.9% saline solution and all lumen were closed using caps. The catheter was sewed onto the skin and a sterile bandage was used to cover the puncture site. A post-operative chest x-ray was performed in order to further confirm the correct placement of the catheter and to exclude complications such as haematoma or pneumothoraces in the case of a subclavian or internal jugular vein puncture. Heparin was not used in order to maintain the patency of central venous catheters during the observed study period.

**Fig 1:**
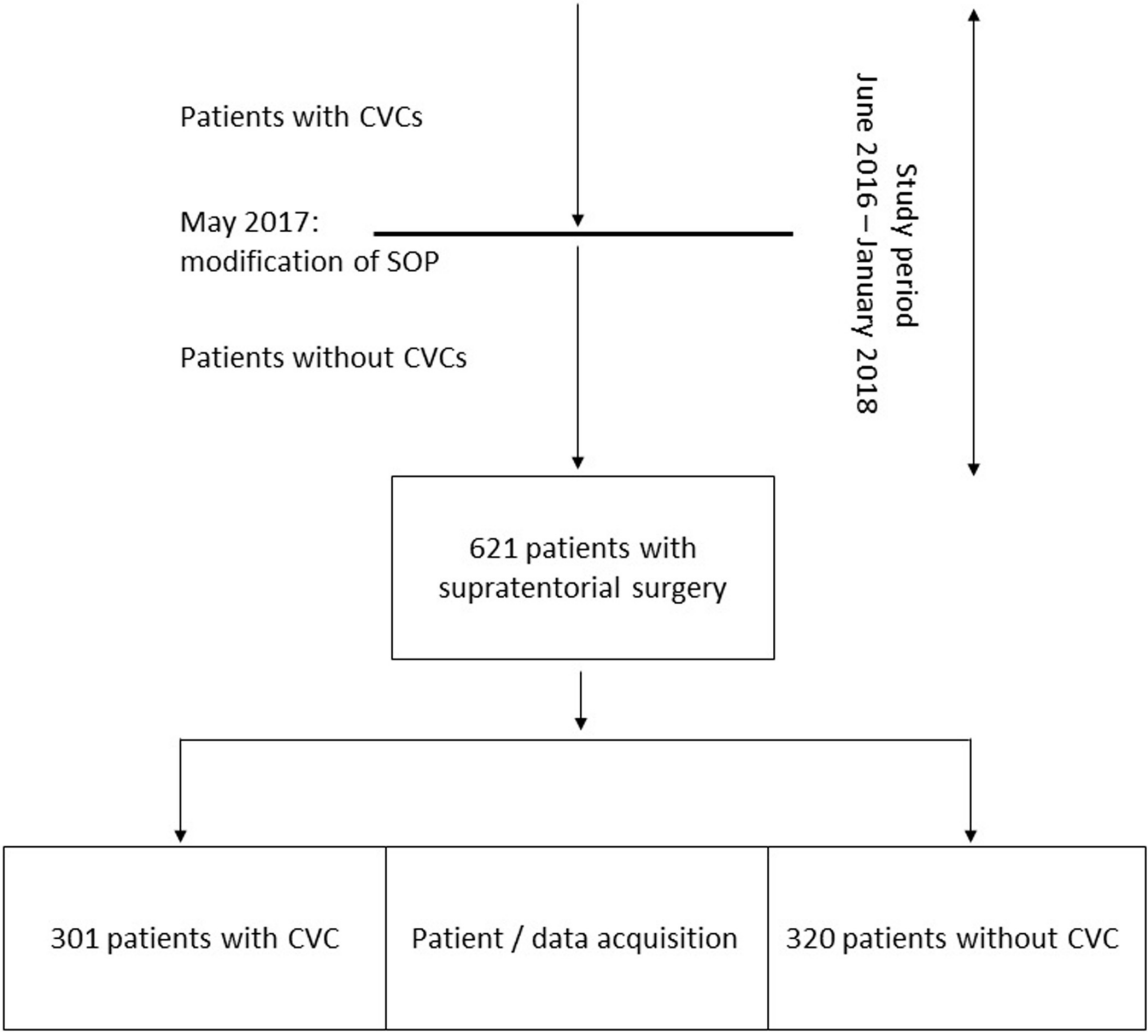
Patient / data acquisition. Retrospective data acquisition was performed between June 2016 and January 2018. Standard anaesthesia procedures were modified during the study period resulting in the termination of obligatory CVC instrumentation for supratentorial procedures. CVC: central venous catheters; SOP: Standard Operating Procedure.

Routine CVC placement in patients undergoing intracranial surgery was terminated in May 2017 as alterations regarding the anaesthesia Standard Operating Procedures were made. This took place independently from the conception of this study. Patients with CVC were therefore included in 2016 and partly in 2017. Patients without CVCs were included after the modification of our Standard Operating Procedures was introduced (Fig 1).

Standard in-house treatment procedures were continued after the patient's routine admission into the ICU. Low molecular weight heparin was used six hours after the surgical procedure in order to prevent a thromboembolism. A subsequent transfer to a peripheral ward was pursued according to the in-house Standard Operating Procedures depending on cardiovascular and respiratory stability (without catecholamine support) as well as stable neurological conditions without any symptom progression. It was additionally required for patients to be relatively pain free (NRS < 3) as well as to exhibit normal laboratory parameters.

### Statistical analysis

Data was analysed using SPSS 22.0.0 (SPSS Inc., Chicago, IL, USA). The Kolomogorov-Smirnov test with Lilliefors correction was used to assess deviations from a normal distribution of the data. Descriptive results were conveyed as mean (SD) for parameters for which the assumption of a normal distribution was not rejected, or otherwise as a median (25^th^;75^th^ percentile). Categorical data was recorded as frequency and as relative frequency as a percentage value. The student’s *t*-test for independent samples was performed to compare groups (CVC vs. non CVC) for continuous data in case of unrejected normality. The Mann-Whitney-*U* test was used alternatively. Associations of categorical variables with the group variable (CVC vs. non CVC) were assessed using Pearson’s χ^2^-test. Binary logistic regression analysis was performed in a multiple approach to evaluate the primary outcome (peri-operative AEs) in dependence of CVC, adjusted for age and body mass index, gender, ASA score, and co-morbidity. In order to evaluate the co-morbidity of patients as to four “relevant pre-existing conditions” considered here, a sum score with values in [0, 4] was calculated. The adjusted odds ratio (OR) for AEs in CVC group vs. non CVC is given with the respective p-value and a 95% confidence interval (CI). A value of p<0.05 was determined as statistically significant.

## Results

### Patient characteristics

A total number of 621 patients (301 with CVC and 320 without CVC) were retrospectively analysed in this study. Patient characteristics such as age, gender, body mass index, co-morbidities and ASA score are summarized in Table 1.

**Table 1:** Patients’ characteristics.

Characteristics			
Demographics	Patients with CVC (n = 301)	Patients without CVC (n = 320)	P
Gender (male/female)	141/160	143/177	0.590
Age (yr)	57 (47;67)	57 (45;69)	0.654
ASA physical status classification			0.008
I	4.3% (13)	5.0% (16)	
II	36.5% (110)	49.4% (158)	
III	55.5% (167)	43.4% (139)	
IV	3.7% (11)	2.2% (7)	
Body mass index (kg/m^2^)	24.9 (22.6;28.2)	24.9 (22.4;28.1)	0.964
Relevant pre-existing conditions			
Heart failure	10.3% (31)	9.4% (30)	0.699
Hypertension	33.9% (102)	34.4% (110)	0.876
Diabetes mellitus	8.0% (24)	5.6% (18)	0.245
Pulmonary diseases (COPD, Asthma)	11.6% (35)	9.7% (31)	0.433

Continous data is presented as mean (SD) or median (25^th^ percentile;75^th^ percentile) depending on the type of data distribution. Categorical data is presented as a percentage value (frequency). ASA = American Society of Anesthesiologists Classification.

The majority of patient characteristics previously mentioned did not differ significantly between groups. However, the ASA classification did demonstrate a significant disparity between groups (p = 0.008). Patients in the CVC group were less frequently classified as ASA II (36.5% (110) vs. 49.4% (158)), and more frequently as ASA III (55.5% (167) vs. 43.4% (139)) compared to patients in the non CVC group. All patients underwent elective intracranial supratentorial procedures (e.g. brain surgery for meningioma, glioma, intracranial metastases) which are shown in detail in Table 2.

**Table 2:** Patients undergoing intracranial supratentorial surgery.

	Patients with CVC (n = 301)	Patients without CVC (n = 320)	P
Supratentorial neurosurgical procedure			0.727
Meningioma	28.9% (87)	29.7% (95)	
Glioma	44.2% (133)	40.3% (129)	
Cavernoma	1.3% (4)	1.9% (6)	
Hippocampectomy / multilobar resection	4.3% (13)	3.4% (11)	
Metastases	17.9% (54)	22.5% (72)	
Arteriovenous malformation	1.3% (4)	0.3% (1)	
Dysplasia	0.7% (2)	0.6% (2)	
Abscess / cyst	1.0% (3)	0.3% (1)	
Lymphoma	0.3% (1)	0.3% (1)	
Sarcoma	0	0.3% (1)	
Pituitary adenoma	0	0.3% (1)	

Data is presented as a percentage value (frequency).

### Primary and secondary endpoints

Our primary endpoint data (peri-operative AEs, Table 3) demonstrates a higher rate of AEs in the CVC group: 81 AEs in the CVC group vs. 51 AEs in the non CVC with comparable group sizes (301 CVC patients vs. 320 non CVC patients). The summary of all observed peri-operative AEs already suggests differences between the two groups. The data demonstrates that cases in which more than one AE was recorded did exist. In detail 69/301 (22.9%) patients with CVC suffered at least from one AE as opposed to 40/320 patients in the non CVC group (12.5%). The corresponding odds of having at least one AE is about twice as high for CVC patients compared to non CVC patients (OR_adjusted_ = 1.98; 95%CI = [1.28, 3.06]; p = 0.002).

**Table 3:** Peri-operative AEs.

	Patients with CVC (n = 301)	Patients without CVC (n = 320)
*Neurological and neurosurgical complications*		
Visual disturbances	12 (4.0%)	6 (1.9%)
Motoric disorders	47 (15.6%)	20 (6.3%)
Sensoric deficits	6 (2.0%)	3 (0.9%)
Hearing deficits	0	1 (0.3%))
Aphasia	5 (1.7%)	4 (1.3%)
Frontal lobe disorder/ right hemisphere syndrome	1 (0.3%)	1 (0.3%)
Intracranial pressure with consciousness impairment	1 (0.3%)	4 (1.3%)
Stroke	2 (0.7%)	1 (0.3%)
Generalized seizure	1 (0.3%)	0
Gerstmann syndrome	0	1 (0.3%)
Perifocal hygroma / Edema	4 (1.3%)	1 (0.3%)
Pneumocephalus	1 (0.3%)	1 (0.3%)
Post-operative bleeding	1 (0.3%)	2 (0.6%)
Re-operation due to bleeding	0	2 (0.6%)
*Cardiovascular Complications*		
Hemodynamic instability	0	1 (0.3%)
Pulmonary embolism	0	1 (0.3%)
*Events of death*	0	2 (0.6%)
Overall AEs (total n = 132)	81	51

Data is presented as frequency (percentage value relative to the number of patients per group.)

Secondary endpoints such as ICU treatment time did not demonstrate any significant differences between either study group (22 (19;24) vs. 21 (19;24) hours, p = 0.413). Additionally, other endpoint data such as ICU ventilation time or surgery duration also did not demonstrate any relevant differences (Table 4).

**Table 4:** Differences in clinical outcomes, treatment and laboratory parameters.

	Patients with CVC (n = 301)	Patients without CVC (n = 320)	P
Treatment on ICU [h]	22 (19;24)	21 (19;24)	0.413
Total time of ventilation [min]	350 (300;440)	335 (281;405)	0.003
Ventilation time on ICU [min]	105 (80;148)	95 (76;139)	0.150
Anaesthesia induction duration [min]	40 (35;50)	30 (25;35)	<0.001
Surgery duration [min]	175 (130;228)	170 (135;215)	0.433
Post-operative antibiotic treatment	31 (10.3%)	23 (7.2%)	0.169
Hospital Stay [days]	9 (7;13)	8 (7;11)	0.210
Post-operative instrumentation with CVC	0	3 (0.9%)	0.092
CRP on admission to ICU	4 (4;4)	4 (4;4)	0.133
CRP day 1 [mg/l]	15 (7;30)	16 (7;28)	0.903
CRP day 2 [mg/l]	88.8 (60.9)	69.3 (57.8)	0.097
CRP day 3 [mg/l]	64.9 (46.8)	79.6 (45.7)	0.274
Leukocytes on admission to ICU	10.2 (4.4)	10.1 (4.8)	0.917
Leukocytes day 1 [n/nl]	11.8 (9.6;14.3)	11.7 (9.8;14.8)	0.693
Leukocytes day 2 [n/nl]	13.3 (4.6)	12.9 (4.6)	0.640
Leukocytes day 3 [n/nl]	12.5 (4.4)	12.4 (4.5)	0.937

Continuous data is presented as a mean (SD) or median (25^th^ percentile;75^th^ percentile) depending on its distribution. Categorical data is presented as frequency (percentage value relative to the number of patients per group).

**Fig 2:**
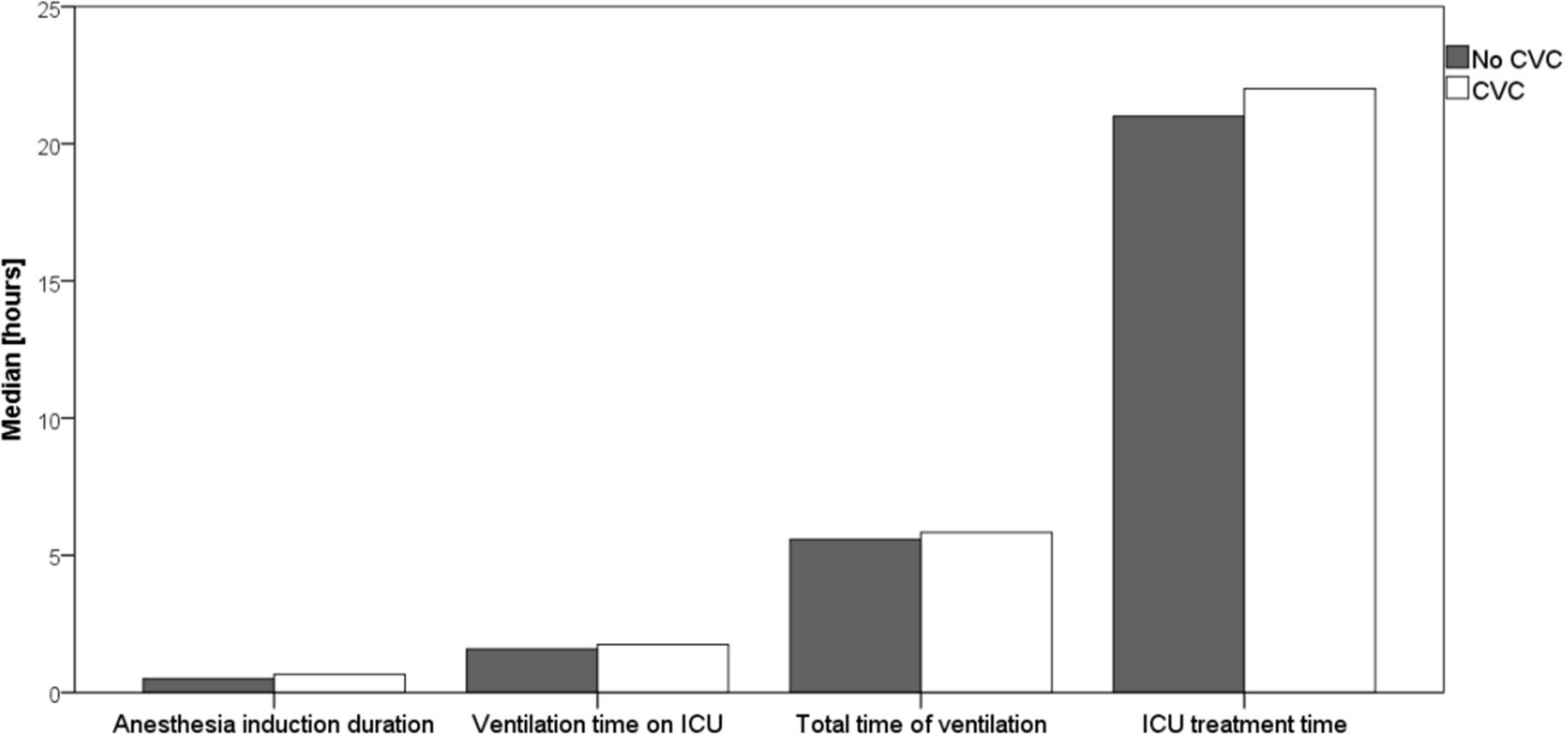
Comparison of induction time, time of ventilation on ICU, total time of ventilation and ICU treatment time period between groups. CVC: Patients with central venous catheters; No CVC: Patients without central venous catheters.

The duration of anaesthesia induction (40 (35;50) vs. 30 (25;35) min, p<0.001) and total ventilation time (350 (300;440) vs. 335 (281;405) min, p<0.003) was observed to be significantly prolonged in the CVC group (Table 4, Fig 2).

No notable differences were otherwise noted in the inflammatory parameters (CRP and leukocytes), post-operative antibiotic treatment or hospital stay (Table 4). In-hospital mortality was found to be 0.32% (2 patients) in the group without CVC owing to a complex and problematic post-operative course. One patient suffered from a pulmonary embolism resulting in a loss of consciousness as well as bradycardia and hypotension after a routine surgical meningioma resection. This lead to a cardiovascular shock which required an intensive cardiopulmonary resuscitation (CPR). The patient was successfully resuscitated and was then supplied with a CVC in order to facilitate the continuous administration of catecholamines. A cCT (cranial computed tomography scan) showed considerable hypoxic and ischemic brain damage despite the successful resuscitation. The patient did not recover and eventually succumbed to irreversible brain damage.

The second patient underwent a bifrontal glioblastoma resection. A pre-operative cCT scan showed a slight midline deviation, causing a mild supratentorial herniation. No complications were observed during the procedure and the patient was promptly extubated. The patient required an emergency cCT due to sudden hemiplegia. The scan showed considerable hemorrhaging into the frontal cortex and a massive supratentorial cerebral edema. This in turn caused a dramatic midline shift which subsequently lead to the compression of the lateral ventricles. The patient was reintubated and an intracranial probe was placed in order to monitor intracranial pressure. The patient was ultimately diagnosed as brain dead, probably due to the massive increase in intracranial pressure (>100 mmHg). A CVC was placed in order to optimally preserve the patient's organs during the ICU stay in order to enable any potential organ donations.

### CVC access and complications

The preferred location of CVC placement in 79.1% of the cases was observed to be the internal jugular vein as opposed to 17.9% (external jugular vein), 2.0% (femoral vein) and 1.0% (subclavian vein). CVC related AEs such as arterial malpuncture (2 patients, 0.7%) and unsuccessful venous puncture (27 patients, 9.0%) were recorded. A single patient (0.3%) was recorded to have suffered from a pneumothorax (Table 5).

**Table 5:** CVC placement and its associated complications.

CVC placement and associated complications	
CVC placement	
Internal jugular vein	238 (79.1%)
External jugular vein	54 (17.9%)
Femoral vein	6 (2.0%)
Subclavian vein	3 (1.0%)
Complications during CVC placement	
Arterial malpuncture	2 (0.7%)
Venous malpuncture	27 (9.0%)
Pneumothorax	1 (0.3%)

Data is presented as frequency (percentage value relative to the number of patients per group).

## Discussion

This retrospective analysis seeks to answer the question whether the use of CVCs provides any clinical advantages in elective intracranial supratentorial surgeries. Patients with CVCs were compared to patients without CVCs in terms of peri-operative AEs. The results of this retrospective study demonstrate that patients with CVCs do not have clinical benefits. Additionally, they are also almost double as likely to be affected by AEs in comparison to patients without CVCs undergoing intracranial supratentorial procedures.

CVC instrumentation in neurosurgical procedures is still a matter of debate at a national and international level. There is no current evidence that neither denies nor confirms any benefit for patients obtaining a CVC as opposed to those that do not. It is common for patients undergoing intracranial surgery to receive an arterial catheter [13] in order to ensure adequate blood pressure monitoring which also enables arterial blood gas analysis. These are essential in order to monitor arterial CO_2_ levels as the arterial-end-tidal CO_2_ gradient has been observed to fluctuate severely.[16] However, the implementation of CVCs varies according to different neurosurgical institutions [14] due to the doubtful benefits and general controversy. Traditionally, central venous pressure has been used as a parameter for fluid management.[17, 18] Central venous pressure is nowadays considered to be an unreliable parameter for measuring intravascular fluid volume.[19] Regardless, CVCs are still implemented for hemodynamic monitoring.[2] This instrumentation can facilitate the acquisition of blood samples in order to record central venous oxygen saturation, which is particularly useful in order to monitor patients with pre-existing cardiovascular conditions. This might enable a guided approach to vasoconstrictor therapy, transfusions and fluid therapy.

This retrospective study aimed to answer the controversial topic whether a CVC should still be considered an adequate and routine tool implemented in elective intracranial supratentorial surgical procedures. Relevant advantages of CVC use such as a decrease in hospital stay or a reduction in total ventilation time were not observed in this study. On the contrary, patients without a CVC undergo a significantly shorter induction time and show a lower rate of peri-operative AEs. The reason for the higher rate of peri-operative AEs in patients with CVC remains unclear. It is most likely a multifactorial occurrence. A propensity score pair-matching was not used to regulate against imbalances in study groups respective to patient characteristics. Instead, for precise and less biased estimation of the odds ratio we used a common regression model adjusted for multiple patient’s characteristics.[20] Nevertheless, there exists the possibility that the occurrence of AEs in the cohorts could have been influenced by other unknown factors / characteristics which were not included in the regression model. Patients that obtained a CVC demonstrate double likely to be affected by AEs as opposed to non CVC patients which in turn highlights disadvantages for the group with central venous lines. Furthermore, the group with CVCs demonstrated a relevant risk for procedural complications such as arterial malpuncture, multiple venous punctures and pneumothorax. Additionally, patients with CVC had a longer anaesthesia induction duration as well as a prolonged time of ventilation which demonstrated further disadvantages for patients with CVCs.

No significant differences were recorded between the study groups regarding cardiovascular or respiratory pre-existing conditions. Nevertheless, differences in the ASA classification were noticed across the two groups, but data analysis was adjusted for this imbalance. Patients suffering from glial or meningeal tumors, who are usually otherwise healthy and do not have any severe cardiovascular or pulmonary diseases, are frequently classified as ASA III. This is due to the fact that neoplastic diseases are considered to be “severe systemic diseases”. In this case, systemic cardiopulmonary diseases are no determining factor in the determination of ASA score II or III. Some centers consider higher ASA scores (III or higher) or even the surgical procedure to be a relative indication for CVC placement. The authors believe the classical ASA score to be misleading as it is questionable whether CVC placement in neurosurgical patients should be solely dictated by a high ASA score, primarily based on an intracranial neurooncological diagnosis. It seems to be justifiable to implement peripheral venous lines in patients without severe pre-existing cardiovascular conditions for the administration of anaesthetics and moderate catecholamine dosages regardless of their ASA score. This in turn offers them the additional benefit of less invasive instrumentation as opposed to those receiving a CVC. Additionally, extended hemodynamic monitoring such as central venous oxygen saturation (a surrogate parameter for mixed venous oxygen saturation [21]) or parenteral nutrition is deemed unnecessary, as these patients usually do not suffer from severe cardiopulmonary pre-existing conditions and usually demonstrate uneventful intra- and / or post-operative developments. In this day and age minimal invasive strategies and fast track surgery with overnight ICU stays are fairly common and clear clinical indications should be set in order to justify a CVC placement in order to prevent unnecessary risks and complications. Less invasive and novel instrumentation methods should attain preference over central venous lines.[10]

Two singular fatalities were observed during the study period in the non CVC group. These deaths were most likely not related to the lack of CVC instrumentation, which is supported by their particular post-operative development and AEs. The first patient suffered a pulmonary embolism which resulted in severe hypoxic ischemic brain damage. The second was pronounced brain dead, probably due to a massive increase in intracranial pressure after an uneventful procedure and an unproblematic extubation in the ICU. Both patients required post-operative CVC insertion in order to facilitate further treatments. Thus, this suggests a clinical benefit of CVC implementation in complex and problematic cases. It should not be routinely implemented for any and all patients. This form of instrumentation warrants specific indications but should be implemented individually, depending on specific patient characteristics and other relevant data.

This study certainly has some limitations. Due to its retrospective design, important issues such as peri-operative blood pressure fluctuations were impossible to determine. This parameter could have potentially been higher in the non CVC group as the modification of catecholamine dosage takes significantly longer if administered via a peripheral line. This might pose a significant disadvantage, as a continuous and stable blood pressure is considered to be beneficial for patients. A prospective study is therefore necessary in order to appropriately assess the impact of this important parameter. Owing to its retrospective nature of this study, there might be further confounders (additional to those that were adjusted for) such as the diversity of the specific intracranial pathology, as well as the variety of surgeons with different areas / levels of expertise. This could have also influenced multiple parameters that affect the occurrence of peri-operative AEs. Additionally, this study was designed to be a retrospective pilot study. The results obtained in this study should be interpreted with care according to this study design. A randomized prospective cross-over study would be desirable in order to confirm our findings.

## Conclusions

Our data demonstrates a lack of significant clinical benefits of CVC use in elective supratentorial neurosurgical procedures and even suggests disadvantages of CVC use with respect to AEs. CVCs should only be placed if patient and case-specific indications exist in order to avoid complications.

## Supporting information

S1 File Data set. Data set necessary to replicate the study findings.
(PDF)
