# Supplementary material for: Retrospective analysis of central venous catheters in elective intracranial surgery - Is there any benefit?
Source: PLoS One. 2019 Dec 19;14(12):e0226641. doi: 10.1371/journal.pone.0226641 (PMC6922467; doi:10.1371/journal.pone.0226641)
Supplement: S1 File — Data set necessary to replicate the study findings. (PDF) [file pone.0226641.s001.pdf]

Group	Sex	Age	Weight	Height	BodyMassIndex	ASA	[illegible]	CVC_femoralis	CVC_jugularis_externa	CVC_jugularis_interna	CVC_subclavia	Puncture_A._carotis	Malpuncture_venous	Wire_problem	Heartfailure	Hypertonus	DM	Pulmonary_Disease	Anesthesia_Induction_Time	Duration_For_Surgery	Time_of_Ventilation_ICU	Overall_Time_Of_Ventilation	ICU_Treatment_Time	Motoric_Disorder	Visual_Disorder	Aphasia	Sensitivity	[illegible]	Perifocal_Edema_Hygroma	Postoperative_Bleeding	Pneumocephalus	[illegible]	Gerstmann_Syndrome	Exitus_Letalis	Hearing_Deficit	Resurgery	Frontal lobe disorder/ right hemisphere syndrome	Postoperative_CVC_on_ICU	Intracranial_Pressure	Cardipulmonary_Instability	Pulmonary_Embolism	Pneumothorax_after_CVC	CRP_Day_0	[illegible]	CRP_Day_2	CRP_Day_3	Leukocytes_Day_0	Leukocytes_Day_1	Leukocytes_Day_2	Leukocytes_Day_3	Antibiotic_Therapy	Hospital_stay
1	1	63,00	67,00	155,00	27,88	3	1	0	0	1	0	0	0	0	0	0	0	0	45	160	100	335	23	0	0	0	0	0	0	0	0	0	0	0	0	0	0	0	0	0	0,00	0	6,00	4,00	38,00	#NULL!	8,90	15,00	13,50	#NULL!	0	9
1	2	63,00	85,00	172,00	24,69	3	2	0	0	1	0	0	0	0	0	1	0	0	50	430	140	655	90	0	0	0	0	0	0	0	0	0	0	0	0	0	0	0	0	0	0,00	0	21,00	96,00	#NULL!	#NULL!	16,20	18,10	#NULL!	#NULL!	0	15
1	1	75,00	65,00	165,00	23,87	3	2	0	0	1	0	0	0	0	0	1	0	1	35	170	200	415	26	1	1	0	0	0	0	0	0	0	0	0	0	0	0	0	0	0	0,00	0	4,00	47,00	96,00	#NULL!	10,30	13,80	13,90	#NULL!	1	17
1	1	38,00	46,00	157,00	18,66	1	2	0	0	1	0	0	0	0	0	0	0	0	50	75	260	405	23	0	0	0	0	0	0	0	0	0	0	0	0	0	0	0	0	0	0,00	0	4,00	18,00	#NULL!	#NULL!	5,80	8,00	#NULL!	#NULL!	0	4
1	1	33,00	47,00	169,00	17,26	3	2	0	0	1	0	0	0	0	0	0	0	0	40	205	135	385	26	0	0	0	0	0	0	0	0	0	0	0	0	0	0	0	0	0	0,00	0	4,00	4,00	24,00	#NULL!	7,50	8,60	13,10	#NULL!	0	8
1	1	21,00	50,00	151,00	21,92	3	4	0	0	1	0	0	0	0	0	0	0	0	85	180	120	385	22	0	0	0	0	0	0	0	0	0	0	0	0	0	0	0	0	0	0,00	0	4,00	22,00	#NULL!	#NULL!	9,20	10,70	#NULL!	#NULL!	0	7
1	1	49,00	95,00	167,00	34,06	2	1	0	0	1	0	0	0	0	0	0	0	0	55	125	75	265	22	0	0	0	0	0	0	0	0	0	0	0	0	0	0	0	0	0	0,00	0	9,00	34,00	#NULL!	#NULL!	8,50	12,50	#NULL!	#NULL!	0	7
1	1	68,00	85,00	167,00	30,47	3	1	0	0	1	0	0	0	0	0	0	0	0	30	315	120	330	12	0	0	0	0	0	0	0	0	0	0	0	0	0	0	0	0	0	0,00	0	4,00	6,00	14,00	#NULL!	22,90	20,30	13,80	#NULL!	0	9
1	1	59,00	60,00	166,00	21,77	1	1	0	0	1	0	0	0	0	0	0	0	0	55	385	40	485	20	0	0	0	0	0	0	0	0	0	0	0	0	0	0	0	0	0	0,00	0	12,00	80,00	235,00	#NULL!	8,40	14,20	10,00	#NULL!	0	7
1	2	59,00	82,00	177,00	26,17	3	2	0	0	1	0	1	0	0	0	1	0	0	75	190	130	390	20	1	0	0	0	0	0	0	0	0	0	0	0	0	0	0	0	0	0,00	0	4,00	15,00	#NULL!	#NULL!	8,80	12,70	#NULL!	#NULL!	0	7
1	1	64,00	91,00	167,00	32,62	3	2	0	1	0	0	0	0	0	0	0	0	1	80	105	80	290	96	0	0	0	0	0	0	0	0	0	0	0	0	0	0	0	0	0	0,00	0	4,00	35,00	32,00	21,00	16,90	15,20	13,90	11,60	0	16
1	1	54,00	65,00	175,00	22,55	2	2	0	1	0	0	0	0	0	0	0	0	0	25	90	60	195	15	0	0	0	0	0	0	0	0	0	0	0	0	0	0	0	0	0	0,00	0	4,00	4,00	#NULL!	#NULL!	7,00	14,20	#NULL!	#NULL!	0	6
1	1	61,00	70,00	170,00	24,22	2	2	0	0	1	0	0	0	0	0	1	0	0	50	160	85	305	23	0	0	0	0	0	0	0	0	0	0	0	0	0	0	0	0	0	0,00	0	4,00	32,00	#NULL!	#NULL!	6,70	8,50	#NULL!	#NULL!	0	6
1	1	58,00	63,00	170,00	21,79	2	1	0	0	1	0	0	0	0	0	1	0	0	45	190	120	310	18	0	0	0	0	0	0	0	0	0	0	0	0	0	0	0	0	0	0,00	0	4,00	7,00	#NULL!	#NULL!	5,90	11,00	#NULL!	#NULL!	0	5
1	2	75,00	128,00	168,00	45,35	4	2	1	0	0	0	0	0	0	0	1	0	1	65	105	250	410	20	0	0	0	0	0	0	0	0	0	0	0	0	0	0	0	0	0	0,00	0	4,00	4,00	#NULL!	#NULL!	12,00	10,00	#NULL!	#NULL!	1	21
1	2	84,00	90,00	190,00	24,93	3	1	0	0	1	0	0	0	0	0	1	0	0	105	110	125	300	16	1	0	0	0	0	0	0	0	0	0	0	0	0	0	0	0	0	0,00	0	4,00	4,00	#NULL!	#NULL!	9,50	12,00	#NULL!	#NULL!	0	22
1	2	18,00	72,00	178,00	22,72	3	2	0	0	1	0	0	1	0	0	0	0	0	35	140	210	410	23	0	0	0	0	0	0	0	0	0	0	0	0	0	0	0	0	0	0,00	0	4,00	17,00	#NULL!	#NULL!	3,70	9,80	#NULL!	#NULL!	0	10
1	1	44,00	55,00	160,00	21,48	2	1	0	0	1	0	0	0	0	0	0	0	1	40	75	79	244	20	0	0	0	0	0	0	0	0	0	0	0	0	0	0	0	0	0	0,00	0	4,00	16,00	#NULL!	#NULL!	6,90	9,60	#NULL!	#NULL!	0	6
1	2	48,00	64,00	174,00	21,13	2	2	0	0	1	0	0	0	0	0	0	0	0	35	130	70	240	17	0	0	0	0	0	0	0	0	0	0	0	0	0	0	0	0	0	0,00	0	4,00	7,00	#NULL!	#NULL!	11,80	22,70	#NULL!	#NULL!	1	13
1	1	66,00	90,00	174,00	29,72	3	5	0	0	1	0	0	0	0	0	0	0	0	40	170	94	369	22	0	0	0	0	0	0	0	0	0	0	0	0	0	0	0	0	0	0,00	0	4,00	4,00	#NULL!	#NULL!	14,60	19,50	#NULL!	#NULL!	0	13
1	1	59,00	78,00	159,00	30,85	2	2	0	0	1	0	0	1	0	1	0	0	0	55	155	90	295	24	0	0	0	0	0	0	0	0	0	0	0	0	0	0	0	0	0	0,00	0	4,00	88,00	161,00	108,00	11,20	14,10	16,10	18,60	0	7
1	1	52,00	56,00	180,00	17,28	3	5	0	0	1	0	0	0	0	0	0	0	0	50	165	130	385	20	0	0	0	0	0	0	0	0	0	0	0	0	0	0	0	0	0	0,00	0	4,00	4,00	#NULL!	#NULL!	7,20	12,20	#NULL!	#NULL!	0	6
1	2	68,00	83,00	170,00	28,71	3	5	0	0	1	0	0	0	0	0	1	1	0	75	205	482	737	39	0	0	1	0	0	0	0	0	0	0	0	0	0	0	0	0	0	0,00	0	110,00	121,00	77,00	#NULL!	6,10	3,80	4,00	#NULL!	0	18
1	2	28,00	85,00	185,00	24,83	3	4	0	0	1	0	0	1	0	0	0	0	0	65	175	60	290	26	0	0	0	0	0	0	0	0	0	0	0	0	0	0	0	0	0	0,00	0	4,00	59,00	#NULL!	#NULL!	6,90	11,60	#NULL!	#NULL!	0	8
1	1	52,00	85,00	180,00	26,23	2	1	0	0	1	0	0	1	0	0	0	0	0	40	240	75	370	23	0	0	0	0	0	0	0	0	0	0	0	0	0	0	0	0	0	0,00	0	4,00	11,00	#NULL!	#NULL!	12,60	14,20	#NULL!	#NULL!	0	8
1	2	66,00	90,00	165,00	33,05	3	1	0	0	1	0	0	0	1	0	1	0	0	65	100	140	350	20	1	0	0	0	0	0	0	0	0	0	0	0	0	0	0	0	0	0,00	0	4,00	34,00	#NULL!	#NULL!	10,30	11,80	#NULL!	#NULL!	0	6
1	1	51,00	78,00	173,00	26,06	1	1	0	0	1	0	0	0	1	0	0	0	0	45	260	50	370	22	0	1	0	0	0	0	0	0	0	0	0	0	0	0	0	0	0	0,00	0	#NULL!	17,00	88,00	#NULL!	#NULL!	15,20	14,40	#NULL!	0	6
1	1	60,00	78,00	160,00	30,46	1	1	0	0	1	0	0	0	0	0	0	0	0	60	220	105	385	23	0	0	0	0	0	0	0	0	0	0	0	0	0	0	0	0	0	0,00	0	4,00	51,00	#NULL!	#NULL!	11,70	7,80	#NULL!	#NULL!	0	8
1	1	61,00	71,00	172,00	23,99	3	5	0	1	0	0	0	0	0	1	1	0	1	25	90	90	270	20	1	0	0	0	0	0	0	0	0	0	0	0	0	0	0	0	0	0,00	0	6,00	8,00	#NULL!	#NULL!	5,90	8,90	#NULL!	#NULL!	0	24
1	2	51,00	84,00	197,00	21,64	2	1	0	0	1	0	0	0	0	0	1	0	0	35	260	130	470	20	0	0	0	0	0	0	0	0	0	0	0	0	0	0	0	0	0	0,00	0	#NULL!	#NULL!	#NULL!	#NULL!	5,50	8,60	#NULL!	#NULL!	0	4
1	1	63,00	55,00	171,00	18,90	1	2	0	0	1	0	0	0	0	0	0	0	0	35	130	65	260	17	0	0	0	0	0	0	0	0	0	0	0	0	0	0	0	0	0	0,00	0	#NULL!	#NULL!	#NULL!	#NULL!	9,90	11,90	#NULL!	#NULL!	0	8

[illegible]

2	1	72,00	65,00	164,00	24,16	3	5	0	0	0	0	0	0	0	0	1	0	0	15	95	75	215	21	0	0	0	0	0	0	0	0	0	0	0	0	0	0	0	0	0	0,00	0	13,00	25,00	#NULL!	#NULL!	11,70	11,70	#NULL!	#NULL!	0	11
2	2	82,00	57,00	170,00	19,72	3	5	0	0	0	0	0	0	0	0	0	0	0	20	195	250	520	21	0	0	0	0	0	0	0	0	0	0	0	0	0	0	0	0	0	0,00	0	4,00	22,00	#NULL!	#NULL!	14,70	10,00	#NULL!	#NULL!	1	23
2	1	14,00	54,00	166,00	19,59	1	2	0	0	0	0	0	0	0	0	0	0	0	35	235	60	330	22	0	0	0	0	0	0	0	0	0	0	0	0	0	0	0	0	0	0,00	0	20,00	31,00	#NULL!	#NULL!	10,60	10,20	#NULL!	#NULL!	0	11
2	1	81,00	90,00	172,00	30,42	3	2	0	0	0	0	0	0	0	0	1	0	0	20	200	225	480	18	0	0	0	0	0	0	0	0	0	0	0	0	0	0	0	0	0	0,00	0	14,00	29,00	#NULL!	#NULL!	9,00	9,50	#NULL!	#NULL!	0	6
2	1	82,00	60,00	168,00	21,25	3	4	0	0	0	0	0	0	0	1	0	0	0	35	210	215	470	23	0	0	0	0	0	0	0	0	0	0	0	0	0	0	0	0	0	0,00	0	4,00	31,00	144,00	89,00	7,10	11,60	10,60	8,00	0	13
2	1	31,00	97,00	170,00	33,56	2	2	0	0	0	0	0	0	0	0	0	0	0	20	145	70	325	19	0	0	0	0	0	0	0	0	0	0	0	1	0	0	0	0	0	0,00	0	4,00	23,00	#NULL!	#NULL!	6,00	12,60	#NULL!	#NULL!	0	4
2	2	56,00	86,00	182,00	25,96	3	2	0	0	0	0	0	0	0	1	0	0	0	35	270	80	390	23	0	0	0	0	0	0	0	0	0	0	0	0	0	0	0	0	0	0,00	0	4,00	37,00	#NULL!	#NULL!	14,10	14,50	#NULL!	#NULL!	0	16
2	2	58,00	120,00	210,00	27,21	2	2	0	0	0	0	0	0	0	0	1	0	0	20	215	210	520	14	0	0	0	0	0	0	0	0	0	0	0	0	0	0	0	0	0	0,00	0	#NULL!	8,00	#NULL!	#NULL!	5,60	11,50	#NULL!	#NULL!	0	9
2	2	80,00	73,00	177,00	23,30	3	13	0	0	0	0	0	0	0	0	1	0	0	20	240	95	355	21	0	0	0	0	0	0	0	0	0	0	0	0	0	0	0	0	0	0,00	0	4,00	16,00	118,00	94,00	9,80	9,00	10,60	16,40	1	40
2	1	83,00	58,00	157,00	23,53	3	5	0	0	0	0	0	0	0	1	1	0	0	25	155	90	285	23	0	0	0	0	0	0	0	0	0	0	0	0	0	0	0	0	0	0,00	0	4,00	8,00	#NULL!	#NULL!	15,00	18,70	#NULL!	#NULL!	0	12
2	1	51,00	80,00	163,00	30,11	2	1	0	0	0	0	0	0	0	0	0	0	0	35	185	110	335	24	0	0	0	0	0	0	0	0	0	0	0	0	0	0	0	0	0	0,00	0	4,00	58,00	#NULL!	#NULL!	10,60	16,50	#NULL!	#NULL!	0	7
2	1	72,00	65,00	170,00	22,49	2	1	0	0	0	0	0	0	0	0	0	0	0	25	190	375	645	20	0	0	0	0	0	0	0	0	0	0	0	0	0	0	0	0	0	0,00	0	4,00	20,00	#NULL!	#NULL!	7,50	12,30	#NULL!	#NULL!	0	9
2	1	30,00	90,00	182,00	27,17	2	2	0	0	0	0	0	0	0	0	0	0	0	30	200	85	330	23	0	0	0	0	0	0	0	0	0	0	0	0	0	0	0	0	0	0,00	0	11,00	61,00	#NULL!	#NULL!	14,30	18,40	#NULL!	#NULL!	0	5
2	2	74,00	99,00	170,00	34,25	3	5	0	0	0	0	0	0	0	0	0	1	0	40	120	180	340	18	0	0	0	0	0	0	0	0	0	0	0	0	0	0	0	0	0	0,00	0	4,00	4,00	#NULL!	#NULL!	6,60	8,80	#NULL!	#NULL!	0	7
2	2	49,00	102,00	192,00	27,66	3	2	0	0	0	0	0	0	0	0	1	0	0	35	150	100	295	24	0	0	0	0	0	0	0	0	0	0	0	0	0	0	0	0	0	0,00	0	4,00	4,00	#NULL!	#NULL!	8,20	11,20	#NULL!	#NULL!	0	6
2	1	76,00	65,00	174,00	21,46	2	2	0	0	0	0	0	0	0	0	0	0	0	25	145	150	320	21	0	0	0	0	0	0	0	0	0	0	0	0	0	0	0	0	0	0,00	0	4,00	24,00	#NULL!	#NULL!	12,60	6,40	#NULL!	#NULL!	0	8
2	1	82,00	75,00	158,00	30,04	2	1	0	0	0	0	0	0	0	0	1	0	1	35	260	80	380	23	0	0	0	0	0	0	0	0	0	0	0	0	0	0	0	0	0	0,00	0	#NULL!	41,00	#NULL!	#NULL!	#NULL!	7,50	#NULL!	#NULL!	0	10
2	1	66,00	46,00	157,00	18,70	3	2	0	0	0	0	0	0	0	0	1	0	0	45	225	160	460	16	0	0	0	0	0	0	0	0	0	0	0	0	0	0	0	0	0	0,00	0	40,00	68,00	#NULL!	#NULL!	6,50	9,90	#NULL!	#NULL!	0	16
2	2	59,00	80,00	183,00	23,88	2	2	0	0	0	0	0	0	0	1	1	0	0	40	90	100	225	26	0	0	0	0	0	0	0	0	0	0	0	0	0	0	0	0	0	0,00	0	4,00	33,00	#NULL!	#NULL!	6,50	14,20	#NULL!	#NULL!	0	15
2	2	75,00	68,00	171,00	23,25	3	2	0	0	0	0	0	0	0	0	0	0	1	55	170	2660	2930	96	0	0	0	0	0	0	1	0	0	0	0	0	1	0	1	0	1	0,00	0	4,00	12,00	156,00	122,00	18,70	20,20	18,10	22,60	1	38
2	1	57,00	61,00	164,00	22,67	3	2	0	0	0	0	0	0	0	0	1	0	0	30	110	90	260	16	0	0	0	0	0	0	0	0	0	0	0	0	0	0	0	0	0	0,00	0	13,00	15,00	#NULL!	#NULL!	4,90	6,50	#NULL!	#NULL!	0	13
2	1	56,00	58,00	168,00	20,54	3	2	0	0	0	0	0	0	0	0	0	0	0	30	160	125	340	23	0	0	0	0	0	0	0	0	0	0	0	0	0	0	0	0	0	0,00	0	4,00	16,00	#NULL!	#NULL!	13,30	14,20	#NULL!	#NULL!	1	16
2	2	34,00	95,00	183,00	28,36	2	1	0	0	0	0	0	0	0	0	0	0	0	35	145	100	290	23	0	0	0	0	0	0	0	0	0	0	0	0	0	0	0	0	0	0,00	0	4,00	42,00	#NULL!	#NULL!	9,70	11,40	#NULL!	#NULL!	0	11
2	1	43,00	83,00	150,00	36,88	2	1	0	0	0	0	0	0	0	0	0	0	0	35	225	325	595	24	1	0	0	0	0	0	0	0	0	0	0	0	0	0	0	0	0	0,00	0	4,00	31,00	#NULL!	#NULL!	12,60	14,40	#NULL!	#NULL!	0	17
2	2	42,00	60,00	175,00	19,59	3	2	0	0	0	0	0	0	0	0	0	0	0	30	145	75	265	22	0	0	0	0	0	0	0	0	0	0	0	0	0	0	0	0	0	0,00	0	4,00	39,00	#NULL!	#NULL!	10,50	17,20	#NULL!	#NULL!	0	8
2	2	41,00	85,00	182,00	25,66	1	1	0	0	0	0	0	0	0	0	0	0	0	15	245	50	370	7	0	0	0	0	0	0	0	0	0	0	0	0	0	0	0	0	0	0,00	0	21,00	#NULL!	#NULL!	#NULL!	16,00	#NULL!	#NULL!	#NULL!	0	7
2	2	35,00	102,00	179,00	31,80	2	2	0	0	0	0	0	0	0	0	0	0	0	40	180	55	295	16	0	0	0	0	0	0	0	0	0	0	0	0	0	0	0	0	0	0,00	0	4,00	17,00	#NULL!	#NULL!	11,80	22,00	#NULL!	#NULL!	0	9
2	1	61,00	85,00	156,00	24,92	2	5	0	0	0	0	0	0	0	0	0	0	0	35	85	285	405	17	1	0	0	0	0	0	0	0	0	0	0	0	0	0	0	0	0	0,00	0	4,00	6,00	#NULL!	#NULL!	8,10	20,80	#NULL!	#NULL!	0	10
2	1	51,00	64,00	175,00	20,89	2	2	0	0	0	0	0	0	0	0	0	0	0	45	125	95	245	25	0	0	0	0	0	0	0	0	0	0	0	0	0	0	0	0	0	0,00	0	4,00	10,00	#NULL!	#NULL!	13,90	13,40	#NULL!	#NULL!	0	12
2	2	69,00	88,00	178,00	27,77	2	2	0	0	0	0	0	0	0	0	0	0	0	45	205	120	375	18	0	0	0	0	0	0	0	0	0	0	0	0	0	0	0	0	0	0,00	0	4,00	15,00	#NULL!	#NULL!	7,10	10,50	#NULL!	#NULL!	0	6
2	1	75,00	58,00	161,00	22,37	3	2	0	0	0	0	0	0	0	0	1	0	1	40	155	155	350	23	0	0	0	0	0	0	0	0	0	0	0	0	0	0	0	0	0	0,00	0	4,00	20,00	#NULL!	#NULL!	4,70	12,40	#NULL!	#NULL!	0	9
2	2	55,00	87,00	186,00	25,14	3	5	0	0	0	0	0	0	0	0	1	0	1	25	100	95	250	20	0	0	0	0	0	0	0	0	0	0	0	0	0	0	0	0	0	0,00	0	4,00	17,00	#NULL!	#NULL!	19,60	28,00	#NULL!	#NULL!	0	10
2	1	53,00	68,00	168,00	24,09	2	1	0	0	0	0	0	0	0	0	0	0	0	40	275	100	415	21	0	0	0	0	0	0	0	0	0	0	0	0	0	0	0	0	0	0,00	0	4,00	37,00	#NULL!	#NULL!	7,40	7,50	#NULL!	#NULL!	0	7
2	1	64,00	62,00	171,00	21,20	3	2	0	0	0	0	0	0	0	0	0	0	0	35	195	165	445	20	0	0	0	0	0	0	0	0	0	0	0	0	0	0	0	0	0	0,00	0	4,00	4,00	#NULL!	#NULL!	6,70	7,70	#NULL!	#NULL!	0	5
2	1	48,00	80,00	170,00	27,68	2	1	0	0	0	0	0	0	0	0	0	0	0	25	165	60	265	16	0	0	0	0	0	0	0	0	0	0	0	0	0	0	0	0	0	0,00	0	4,00	11,00	#NULL!	#NULL!	13,10	11,20	#NULL!	#NULL!	0	6
2	2	39,00	83,00	186,00	23,90	2	4	0	0	0	0	0	0	0	0	0	0	1	25	200	80	340	18	0	0	0	0	0	0	0	0	0	0	0	0	0	0	0	0	0	0,00	0	4,00	34,00	#NULL!	#NULL!	6,70	10,60	#NULL!	#NULL!	0	6
2	1	40,00	70,00	170,00	24,22	2	2	0	0	0	0	0	0	0	0	0	0	0	35	75	80	200	25	0	0	0	0	0	0	0	0	0	0	0	0	0	0	0	0	0	0,00	0	4,00	7,00	#NULL!	#NULL!	6,20	10,50	#NULL!	#NULL!	0	8
2	1	60,00	74,00	168,00	26,21	2	5	0	0	0	0	0	0	0	0	1	0	0	30	150	80	265	18	0	0	0	0	0	0	0	0	0	0	0	0	0	0	0	0	0	0,00	0	4,00	4,00	#NULL!	#NULL!	5,30	9,80	#NULL!	#NULL!	0	8
2	2	54,00	104,00	185,00	30,38	2	4	0	0	0	0	0	0	0	0	0	0	0	25	250	85	390	17	0	0	0	0	0	0	0	0	0	0	0	0	0	0	0	0	0	0,00	0	4,00	14,00	#NULL!	#NULL!	10,50	15,40	#NULL!	#NULL!	0	7
2	2	58,00	76,00	187,00	21,73	3	2	0	0	0	0	0	0	0	1	1	0	0	55	250	175	465	22	1	0	0	0	0	0	0	0	0	0	0	0	0	0	0	0	0	0,00	0	7,00	11,00	#NULL!	#NULL!	9,90	7,50	#NULL!	#NULL!	0	6
2	1	54,00	62,00	163,00	23,33	2	5	0	0	0	0	0	0	0	0	0	0	0	20	85	75	190	21	0	0	0	0	0	0	0	0	0	0	0	0	0	0	0	0	0	0,00	0	4,00	14,00	#NULL!	#NULL!	10,20	11,40	#NULL!	#NULL!	0	18
2	1	55,00	61,00	173,00	20,38	2	2	0	0	0	0	0	0	0	0	0	0	0	40	145	755	940	44	1	0	0	0	0	0	0	0	0	0	0	0	0	0	0	0	0	0,00	0	4,00	4,00	7,00	#NULL!	8,30	9,40	7,70	#NULL!	0	10
2	1	39,00	76,00	178,00	23,98	2	2	0	0	0	0	0	0	0	0	0	0	0	30	135	75	260	21	0	0	0	0	0	0	0	0	0	0	0	0	0	0	0	0	0	0,00	0	4,00	4,00	#NULL!	#NULL!	3,90	6,60	#NULL!	#NULL!	0	5
2	1	47,00	69,00	160,00	26,95	3	5	0	0	0	0	0	0	0	0	0	0	0	45	105	150	285	24	0	0	0	0	0	0	0	0	0	0	0	0	0	0	0	0	0	0,00	0	4,00	11,00	#NULL!	#NULL!	8,80	11,20	#NULL!	#NULL!	0	7
2	1	46,00	110,00	176,00	35,50	3	1	0	0	0	0	0	0	0	0	1	0	0	30	275	90	435	22	0	0	0	0	0	0	0	0	0	0	0	0	0	0	0	0	0	0,00	0	4,00	13,00	#NULL!	#NULL!	10,00	16,30	#NULL!	#NULL!	0	6
2	1	65,00	55,00	162,00	20,95	2	5	0	0	0	0	0	0	0	0	0	0	0	25	120	225	385	16	0	0	0	0	0	0	0	0	0	0	0	0	0	0	0	0	0	0,00	0	14,00	17,00	#NULL!	#NULL!	5,90	7,40	#NULL!	#NULL!	0	6
2	1	71,00	68,00	160,00	26,56	3	2	0	0	0	0	0	0	0	0	1	0	0	45	195	260	555	21	0	0	0	0	0	0	0	0	0	0	0	0	0	0	0	0	0	0,00	0	4,00	4,00	#NULL!	#NULL!	7,20	14,70	#NULL!	#NULL!	0	8
2	1	46,00	75,00	175,00	24,48	3	2	0	0	0	0	0	0	0	0	0	0	0	35	115	105	280	23	0	0	0	0	0	0	0	0	0	0	0	0	0	0	0	0	0	0,00	0	4,00	4,00	#NULL!	#NULL!	5,20	8,00	#NULL!	#NULL!	0	4
2	2	60,00	95,00	180,00	29,32	3	2	0	0	0	0	0	0	0	0	0	0	0	20	160	140	405	20	0	0	0	0	0	0	0	0	0	0	0	0	0	0	0	0	0	0,00	0	4,00	4,00	#NULL!	#NULL!	5,90	9,60	#NULL!	#NULL!	0	7
2	1	28,00	61,00	179,00	19,03	2	2	0	0	0	0	0	0	0	0	0	0	0	40	155	70	300	24	0	0	0	0	0	0	0	0	0	0	0	0	0	0	0	0	0	0,00	0	4,00	11,00	#NULL!	#NULL!	6,20	10,50	#NULL!	#NULL!	0	13
2	2	37,00	84,00	175,00	27,42	3	2	0	0	0	0	0	0	0	0	0	0	0	45	140	85	270	20	0	0	0	0	0	0	0	0	0	0	0	0	0	0	0	0	0	0,00	0	4,00	20,00	#NULL!	#NULL!	6,70	8,30	#NULL!	#NULL!	0	8
2	2	71,00	77,00	180,00	23,76	3	5	0	0	0	0	0	0	0	0	0	0	0	25	160	80	305	16	0	0	0	0	0	0	0	0	0	0	0	0	0	0	0	0	0	0,00	0	4,00	4,00	#NULL!	#NULL!	13,20	9,20	#NULL!	#NULL!	0	8
2	1	26,00	70,00	174,00	23,12	2	9	0	0	0	0	0	0	0	0	0	0	0	20	150	100	325	24	0	0	0	0	0	0	0	0	0	0	0	0	0	0	0	0	0	0,00	0	4,00	79,00	#NULL!	#NULL!	3,10	3,50	#NULL!	#NULL!	0	9
2	1	52,00	63,00	172,00	21,29	2	5	0	0	0	0	0	0	0	0	0	0	0	25	145	195	375	20	0	0	0	0	0	0	0	0	0	0	0	0	0	0	0	0	0	0,00	0	6,00	7,00	#NULL!	#NULL!	20,60	14,40	#NULL!	#NULL!	1	9
2	2	52,00	90,00	187,00	25,73	3	5	0	0	0	0	0	0	0	0	0	0	0	35	120	105	245	28	0	0	0	0	0	0	0	0	0	0	0	0	0	0	0	0	0	0,00	0	4,00	13,00	#NULL!	#NULL!	12,60	13,80	#NULL!	#NULL!	0	9
2	2	69,00	97,00	178,00	30,61	2	1	0	0	0	0	0	0	0	0	1	0	0	25	260	146	481	21	0	0	0	0	0	0	0	0	0	0	0	0	0	0	0	0	0	0,00	0	4,00	19,00	#NULL!	#NULL!	7,00	11,00	#NULL!	#NULL!	0	6
2	2	77,00	72,00	182,00	21,73	3	2	0	0	0	0	0	0	0	1	1	0	0	30	110	110	275	14	0	0	0	0	0	0	0	0	0	0	0	0	0	0	0	0	0	0,00	0	4,00	4,00	#NULL!	#NULL!	6,30	9,70	#NULL!	#NULL!	0	22
2	2	64,00	69,00	173,00	23,05	2	4	0	0	0	0	0	0	0	0	0	0	0	25	200	145	400	21	0	0	0	0	0	0	0	0	0	0	0	0	0	0	0	0	0	0,00	0	4,00	49,00	178,00	122,00	8,30	13,30	13,30	9,30	0	12
2	1	74,00	56,00	164,00	20,82	2	1	0	0	0	0	0	0	0	0	1	0	0	30	210	210	425	27	0	0	0	0	0	0	0	0	0	0	0	0	0	0	0	0	0	0,00	0	4,00	38,00	#NULL!	#NULL!	4,60	13,60	#NULL!	#NULL!	0	8
2	1	42,00	48,00	152,00	20,77	2	2	0	0	0	0	0	0	0	0	1	0	1	40	165	235	470	20	0	0	0	0	0	0	0	0	0	0	0	0	0	0	0	0	0	0,00	0	4,00	5,00	#NULL!	#NULL!	14,70	24,30	#NULL!	#NULL!	0	12
2	2	66,00	82,00	175,00	26,77	2	1	0	0	0	0	0	0	0	0	1	0	0	40	170	61	256	14	0	0	0	0	0	0	0	0	0	0	0	0	0	0	0	0	0	0,00	0	4,00	4,00	34,00	#NULL!	11,00	14,10	15,70	#NULL!	0	5
2	1	64,00	58,00	168,00	20,54	2	2	0	0	0	0	0	0	0	0	1	0	0	40	150	105	305	18	0	0	0	0	0	0	0	0	0	0	0	0	0	0	0	0	0	0,00	0	#NULL!	4,00	#NULL!	#NULL!	#NULL!	11,00	#NULL!	#NULL!	0	9
2	1	52,00	107,00	168,00	37,91	2	2	0	0	0	0	0	0	0	0	1	0	0	35	200	110	405	21	0	0	0	0	0	0	0	0	0	0	0	0	0	0	0	0	0	0,00	0	10,00	35,00	#NULL!	#NULL!	3,30	7,90	#NULL!	#NULL!	0	7
2	1	48,00	65,00	169,00	22,75	2	1	0	0	0	0	0	0	0	0	0	0	0	30	145	80	270	24	0	0	0	0	0	0	0	0	0	0	0	0	0	0	0	0	0	0,00	0	4,00	24,00	#NULL!	#NULL!	8,10	10,50	#NULL!	#NULL!	1	14
2	1	63,00	66,00	161,00	25,46	3	5	0	0	0	0	0	0	0	0	1	0	0	20	165	60	245	18	0	0	0	0	0	0	0	0	0	0	0	0	0	0	0	0	0	0,00	0	4,00	9,00	#NULL!	#NULL!	11,60	11,40	#NULL!	#NULL!	0	7
2	2	54,00	84,00	172,00	28,39	2	5	0	0	0	0	0	0	0	0	0	0	0	30	100	45	190	26	0	0	0	0	0	0	0	0	0	0	0	0	0	0	0	0	0	0,00	0	4,00	13,00	#NULL!	#NULL!	8,00	13,60	#NULL!	#NULL!	0	5
2	2	74,00	76,00	178,00	23,98	3	2	0	0	0	0	0	0	0	0	1	0	0	30	115	185	340	23	0	0	0	0	0	0	0	0	0	0	0	0	0	0	0	0	0	0,00	0	4,00	26,00	#NULL!	#NULL!	15,80	16,10	#NULL!	#NULL!	0	14
2	2	68,00	90,00	176,00	29,05	2	1	0	0	0	0	0	0	0	0	1	0	0	60	170	70	305	23	0	0	0	0	0	0	0	0	0	0	0	0	0	0	0	0	0	0,00	0	4,00	15,00	#NULL!	#NULL!	9,50	10,00	#NULL!	#NULL!	0	6
2	1	54,00	98,00	163,00	36,88	2	1	0	0	0	0	0	0	0	0	0	0	0	15	210	60	305	22	1	0	0	1	0	0	0	0	0	0	0	0	0	0	0	0	0	0,00	0	4,00	8,00	#NULL!	#NULL!	11,80	11,10	#NULL!	#NULL!	0	12
2	2	36,00	82,00	188,00	23,20	3	2	0	0	0	0	0	0	0	0	0	0	0	35	180	55	285	24	0	0	0	0	0	0	0	0	0	0	0	0	0	0	0	0	0	0,00	0	4,00	4,00	#NULL!	#NULL!	7,10	7,40	#NULL!	#NULL!	0	11
2	2	49,00	75,10	182,00	22,67	3	2	0	0	0	0	0	0	0	0	1	0	0	25	140	50	235	21	0	0	0	0	0	0	0	0	0	0	0	0	0	0	0	0	0	0,00	0	4,00	57,00	#NULL!	#NULL!	4,80	5,00	#NULL!	#NULL!	0	7
2	1	66,00	93,00	170,00	32,17	2	1	0	0	0	0	0	0	0	0	1	0	1	25	165	140	390	16	0	0	0	0	0	0	0	0	0	0	0	0	0	0	0	0	0	0,00	0	4,00	10,00	#NULL!	#NULL!	8,10	10,20	#NULL!	#NULL!	0	9
2	2	60,00	82,00	170,00	29,37	2	1	0	0	0	0	0	0	0	0	0	0	0	25	240	40	310	21	0	0	0	0	0	0	0	0	0	0	0	0	0	0	0	0	0	0,00	0	4,00	45,00	#NULL!	#NULL!	8,70	9,10	#NULL!	#NULL!	0	7
2	1	45,00	85,00	175,00	27,75	2	1	0	0	0	0	0	0	0	0	0	0	0	35	180	90	320	17	0	0	0	0	0	0	0	0	0	0	0	0	0	0	0	0	0	0,00	0	4,00	16,00	#NULL!	#NULL!	9,30	7,20	#NULL!	#NULL!	0	8
2	2	31,00	66,00	172,00	22,30	2	4	0	0	0	0	0	0	0	0	0	0	0	30	210	125	380	20	0	0	0	0	0	0	0	0	0	0	0	0	0	0	0	0	0	0,00	0	4,00	24,00	#NULL!	#NULL!	6,00	10,60	#NULL!	#NULL!	0	7
2	2	77,00	72,00	182,00	21,73	3	2	0	0	0	0	0	0	0	1	1	0	0	30	110	1130	1285	26	0	0	0	0	0	0	0	0	0	0	0	0	0	0	0	0	0	0,00	0	24,00	39,00	#NULL!	#NULL!	10,00	7,00	#NULL!	#NULL!	0	22
2	1	68,00	49,00	161,00	18,90	3	2	0	0	0	0	0	0	0	0	1	0	0	25	115	177	322	22	0	0	0	0	0	0	0	0	0	0	0	0	0	0	0	0	0	0,00	0	4,00	25,00	#NULL!	#NULL!	8,80	17,20	#NULL!	#NULL!	0	11
2	1	63,00	105,00	170,00	36,33	3	2	0	0	0	0	0	0	0	0	1	0	0	35	115	50	200	24	0	0	0	0	0	0	0	0	0	0	0	0	0	0	0	0	0	0,00	0	4,00	16,00	#NULL!	#NULL!	8,30	11,40	#NULL!	#NULL!	0	8
2	1	79,00	57,00	160,00	22,26	3	2	0	0	0	0	0	0	0	0	1	0	0	35	175	145	350	21	0	0	0	0	0	0	0	0	0	0	0	0	0	0	0	0	0	0,00	0	4,00	20,00	#NULL!	#NULL!	8,10	11,80	#NULL!	#NULL!	0	11
2	1	73,00	92,00	170,00	31,83	3	1	0	0	0	0	0	0	0	0	0	0	0	35	250	155	485	16	0	0	0	0	0	0	0	0	0	0	0	0	0	0	0	0	0	0,00	0	13,00	14,00	#NULL!	#NULL!	11,00	8,60	#NULL!	#NULL!	0	8
2	2	44,00	77,00	179,00	24,03	3	5	0	0	0	0	0	0	0	0	0	0	0	30	75	80	195	24	0	0	0	0	0	0	0	0	0	0	0	0	0	0	0	0	0	0,00	0	7,00	25,00	#NULL!	#NULL!	6,90	11,90	#NULL!	#NULL!	0	5
2	1	58,00	93,00	158,00	37,25	3	1	0	0	0	0	0	0	0	0	1	1	0	20	285	55	390	19	0	0	0	0	0	0	0	0	0	0	0	0	0	0	0	0	0	0,00	0	4,00	26,00	#NULL!	#NULL!	9,40	15,10	#NULL!	#NULL!	0	8
2	1	31,00	65,00	163,00	24,46	3	5	0	0	0	0	0	0	0	0	0	0	0	50	115	70	250	22	0	0	0	0	0	0	0	0	0	0	0	0	0	0	0	0	0	0,00	0	4,00	20,00	#NULL!	#NULL!	3,60	7,30	#NULL!	#NULL!	0	6
2	1	72,00	60,00	180,00	18,51	3	5	0	0	0	0	0	0	0	0	0	0	1	35	150	110	320	22	0	0	0	0	0	0	0	0	0	0	0	0	0	0	0	0	0	0,00	0	4,00	10,00	#NULL!	#NULL!	13,40	13,20	#NULL!	#NULL!	0	5
2	1	34,00	103,00	167,00	36,93	2	1	0	0	0	0	0	0	0	0	0	0	0	25	390	65	520	19	0	0	0	0	0	0	0	0	0	0	0	0	0	0	0	0	0	0,00	0	8,00	16,00	#NULL!	#NULL!	14,10	11,90	#NULL!	#NULL!	0	8
2	2	35,00	82,00	175,00	26,77	3	2	0	0	0	0	0	0	0	0	0	0	0	45	235	65	345	21	0	0	0	0	0	0	0	0	0	0	0	0	0	0	0	0	0	0,00	0	4,00	75,00	#NULL!	#NULL!	10,20	10,80	#NULL!	#NULL!	0	5
2	2	88,00	70,00	164,00	26,00	3	5	0	0	0	0	0	0	0	1	1	0	0	35	180	130	350	25	0	0	0	0	0	0	0	0	0	0	0	0	0	0	0	0	0	0,00	0	4,00	36,00	#NULL!	#NULL!	9,40	14,00	#NULL!	#NULL!	0	9
2	2	57,00	82,00	180,00	25,30	2	2	0	0	0	0	0	0	0	0	0	0	0	30	180	90	305	24	0	0	0	0	0	0	0	0	0	0	0	0	0	0	1	0	0	0,00	0	4,00	5,00	#NULL!	#NULL!	18,50	11,80	#NULL!	#NULL!	0	9
2	2	48,00	102,00	180,00	31,48	2	1	0	0	0	0	0	0	0	0	1	0	0	20	140	115	320	20	0	0	0	0	0	0	0	0	0	0	0	0	0	0	0	0	0	0,00	0	4,00	4,00	#NULL!	#NULL!	9,10	12,20	#NULL!	#NULL!	0	6
2	1	55,00	55,00	162,00	20,95	2	1	0	0	0	0	0	0	0	0	0	0	0	35	235	45	320	23	0	0	0	0	0	0	0	0	0	0	0	0	0	0	0	0	0	0,00	0	4,00	8,00	#NULL!	#NULL!	5,80	10,70	#NULL!	#NULL!	0	12
2	2	65,00	55,00	180,00	16,97	4	5	0	0	0	0	0	0	0	0	0	0	0	55	95	115	270	21	1	0	0	0	0	0	0	0	0	0	0	0	0	0	0	0	0	0,00	0	23,00	30,00	#NULL!	#NULL!	9,60	6,80	#NULL!	#NULL!	0	11
2	1	69,00	47,00	149,00	21,17	3	5	0	0	0	0	0	0	0	0	0	0	0	35	95	110	235	25	0	0	0	0	0	0	0	0	0	0	0	0	0	0	0	0	0	0,00	0	4,00	52,00	78,00	#NULL!	9,20	16,60	16,80	#NULL!	1	38
2	1	79,00	66,00	156,00	27,12	3	1	0	0	0	0	0	0	0	0	1	0	1	40	200	80	320	25	1	0	0	0	0	0	0	0	0	0	0	0	0	0	0	0	0	0,00	0	4,00	42,00	#NULL!	#NULL!	7,50	14,10	#NULL!	#NULL!	0	8
2	2	55,00	92,00	182,00	27,77	2	1	0	0	0	0	0	0	0	0	0	0	0	35	270	100	405	23	0	0	0	0	0	0	0	0	0	0	0	0	0	0	0	0	0	0,00	0	4,00	11,00	#NULL!	#NULL!	5,10	10,70	#NULL!	#NULL!	0	6
2	1	34,00	60,00	165,00	22,03	2	10	0	0	0	0	0	0	0	0	0	0	0	15	140	135	305	22	0	0	0	0	0	0	0	0	0	0	0	0	0	0	0	0	0	0,00	0	5,00	25,00	#NULL!	#NULL!	7,60	11,50	#NULL!	#NULL!	1	43
2	1	54,00	66,00	160,00	25,80	3	1	0	0	0	0	0	0	0	0	1	0	0	30	160	95	285	19	0	1	0	0	0	0	0	0	0	0	0	0	0	0	0	0	0	0,00	0	4,00	12,00	#NULL!	#NULL!	6,60	8,70	#NULL!	#NULL!	0	6
2	2	48,00	91,00	182,00	27,47	2	1	0	0	0	0	0	0	0	0	0	0	0	35	240	50	340	21	0	0	0	0	0	0	0	0	0	0	0	0	0	0	0	0	0	0,00	0	4,00	#NULL!	#NULL!	#NULL!	8,20	#NULL!	#NULL!	#NULL!	0	12
2	2	49,00	88,00	185,00	25,71	1	2	0	0	0	0	0	0	0	0	0	0	0	25	225	115	380	22	0	0	0	0	0	0	0	0	0	0	0	0	0	0	0	0	0	0,00	0	4,00	23,00	#NULL!	#NULL!	10,50	11,50	#NULL!	#NULL!	0	6
2	2	66,00	92,00	178,00	29,03	2	2	0	0	0	0	0	0	0	0	1	1	0	40	130	85	320	22	0	0	0	0	0	0	0	0	0	0	0	0	0	0	0	0	0	0,00	0	6,00	32,00	#NULL!	#NULL!	11,60	13,60	#NULL!	#NULL!	0	8
2	1	29,00	78,00	163,00	29,35	2	1	0	0	0	0	0	0	0	0	0	0	0	45	120	105	265	18	0	0	0	0	0	0	0	0	0	0	0	0	0	0	0	0	0	0,00	0	4,00	#NULL!	#NULL!	#NULL!	5,50	#NULL!	#NULL!	#NULL!	0	5
2	2	62,00	101,00	170,00	24,94	3	2	0	0	0	0	0	0	0	0	1	0	0	30	255	16510	16805	312	0	0	0	0	0	0	1	0	0	0	0	0	1	0	0	1	0	0,00	0	7,00	54,00	100,00	59,00	7,40	13,50	13,80	10,70	1	14
2	1	45,00	83,00	165,00	20,48	3	1	0	0	0	0	0	0	0	0	0	0	0	50	155	135	315	17	0	0	0	0	0	0	0	0	0	0	0	0	0	0	0	0	0	0,00	0	4,00	9,00	#NULL!	#NULL!	10,90	16,10	#NULL!	#NULL!	0	6
2	1	77,00	73,00	167,00	26,17	2	2	0	0	0	0	0	0	0	0	0	0	0	45	170	73	278	23	0	0	0	0	0	0	0	0	0	0	0	0	0	0	0	0	0	0,00	0	4,00	22,00	66,00	#NULL!	12,40	11,90	12,40	#NULL!	0	14
2	1	67,00	52,00	162,00	19,81	3	1	0	0	0	0	0	0	0	0	1	0	0	25	125	80	275	22	0	0	0	0	0	0	0	0	0	0	0	0	0	0	0	0	0	0,00	0	4,00	18,00	#NULL!	#NULL!	11,40	14,80	#NULL!	#NULL!	0	10
2	1	57,00	65,00	170,00	22,49	2	5	0	0	0	0	0	0	0	0	0	0	0	30	80	140	245	26	1	0	0	1	0	0	0	0	0	0	0	0	0	0	0	0	0	0,00	0	4,00	12,00	#NULL!	#NULL!	17,70	16,50	#NULL!	#NULL!	0	19
2	1	75,00	55,00	168,00	19,48	1	2	0	0	0	0	0	0	0	0	0	0	0	35	105	115	260	24	0	0	0	0	0	0	0	0	0	0	0	0	0	0	0	0	0	0,00	0	#NULL!	13,00	#NULL!	#NULL!	7,40	9,20	#NULL!	#NULL!	0	8
2	2	52,00	73,00	182,00	22,03	2	2	0	0	0	0	0	0	0	0	0	0	0	30	325	80	445	24	1	0	0	0	0	0	0	0	0	0	0	0	0	0	0	0	0	0,00	0	4,00	27,00	#NULL!	#NULL!	8,20	14,90	#NULL!	#NULL!	0	11
2	1	64,00	65,00	174,00	21,46	3	2	0	0	0	0	0	0	0	0	1	0	0	60	180	135	375	16	0	0	0	0	0	0	0	0	0	0	0	0	0	0	0	0	0	0,00	0	4,00	4,00	#NULL!	#NULL!	6,10	4,80	#NULL!	#NULL!	0	9
2	1	68,00	53,00	179,00	18,33	2	1	0	0	0	0	0	0	0	0	0	0	0	35	180	80	295	23	0	0	0	0	0	0	0	0	0	0	0	0	0	0	0	0	0	0,00	0	4,00	14,00	#NULL!	#NULL!	4,90	6,40	#NULL!	#NULL!	0	6
2	2	44,00	97,00	190,00	26,86	3	2	0	0	0	0	0	0	0	0	0	0	0	20	135	90	265	21	0	0	0	0	0	0	0	0	0	0	0	0	0	0	0	0	0	0,00	0	4,00	7,00	#NULL!	#NULL!	7,60	14,10	#NULL!	#NULL!	0	6
2	2	83,00	75,00	175,00	24,48	2	2	0	0	0	0	0	0	0	0	1	0	0	35	205	205	450	16	0	0	0	0	0	0	0	0	0	0	0	0	0	0	0	0	0	0,00	0	4,00	6,00	#NULL!	#NULL!	11,60	11,10	#NULL!	#NULL!	0	26
2	1	31,00	95,00	165,00	34,89	2	2	0	0	0	0	0	0	0	0	0	0	0	35	202	98	325	21	0	0	0	0	0	0	0	0	0	0	0	0	0	0	0	0	0	0,00	0	9,00	26,00	#NULL!	#NULL!	8,50	17,00	#NULL!	#NULL!	0	10
2	1	39,00	64,00	172,00	21,63	2	2	0	0	0	0	0	0	0	0	0	0	0	40	100	90	240	26	0	0	0	0	0	0	0	0	0	0	0	0	0	0	0	0	0	0,00	0	4,00	20,00	#NULL!	#NULL!	7,20	14,10	#NULL!	#NULL!	0	8
2	1	77,00	62,00	165,00	22,77	3	5	0	0	0	0	0	0	0	0	0	0	0	25	150	106	301	16	0	0	0	0	0	0	0	0	0	0	0	0	0	0	0	0	0	0,00	0	4,00	4,00	9,00	#NULL!	13,20	14,30	15,80	#NULL!	0	19
